# Anxiety, depression, psychological stress and coping style in medical postgraduates in southeastern China when restricted to commuting between the campus and hospital during the COVID-19 pandemic

**DOI:** 10.3389/fpsyt.2022.1035075

**Published:** 2023-01-06

**Authors:** Tianci Tan, Wenting Liu, Qianqian Zhao, Yanfei Hou, Yuan Yang, Xiaxin Wu, Yuying Wang, Yu Chen, Guangli Hu

**Affiliations:** ^1^Graduate School of Southern Medical University, Guangzhou, China; ^2^School of Nursing, Southern Medical University, Guangzhou, China; ^3^Guangdong Mental Health Center, Guangdong Provincial People’s Hospital, Guangdong Academy of Medical Sciences, Guangzhou, China; ^4^School of Nursing, Peking University, Beijing, China

**Keywords:** anxiety, depression, psychological stress, coping style, medical postgraduates, COVID-19

## Abstract

**Background:**

As the COVID-19 epidemic was gradually brought under control, a new autumn semester began in 2020. How was the mental health of postgraduates as they experienced quarantine at home, only commuting between the school and hospital?

**Methods:**

The research was conducted in a cross-sectional online survey in October 2020. The data were collected from 1,645 medical postgraduates (master’s and doctoral students) by using the demographic information questionnaire, the Self-rating Depression Scale (SDS), the Self-rating Anxiety Scale (SAS), the Questionnaire on Psychological Stressors of Postgraduates (QPSP), the Simplified Coping Style Questionnaire (SCSQ) and the Social Support Rate Scale (SSRS). One-way ANOVA and Pearson correlation were used to explore the relationships among anxiety, depression, psychological stressors, social support and coping style. Structural equation modeling (SEM) was conducted to assess the mediation model.

**Results:**

Among the total of 1,645 medical postgraduates, 21.6% (*n* = 356) had self-rated depression symptoms, and 9.4% (*n* = 155) had self-rated anxiety symptoms. The main disturbances they experienced were employment, academic and interpersonal pressure. The master of third grade students had the highest employment pressure, and the master of second grade students had the highest academic and interpersonal pressure. Negative coping played a negative mediating role and social support played a positive mediating role in the relationships between perceived stress and anxiety (β = 0.027, *P* < 0.01; β = 0.124, *P* < 0.01) and depression (β = 0.016, *P* < 0.01; β = 0.193, *P* < 0.01).

**Conclusion:**

Medical postgraduates in China restricted to studies on campus and in the hospital experienced psychological distress. Our results suggest that providing employment and learning guidance, while strengthening social support and guiding positive coping may be effective at improving the mental health of the medical graduate students, mediating their perceived stress and negative emotions.

## 1. Introduction

The restriction of several activities, school closures, and the lockdown of cities were measures implemented to cope with the outbreak of COVID-19 (1). Chaos, fear, isolation and social distancing, as well as the uncertainty of the virus, were always present. The sacrifices of medical workers globally finally relieved pressure in the fight against this major public health event (2). In addition to the medical staff, medical students are also an important force in the epidemic prevention and control in the future, so their mental health under the COVID-19 epidemic is also worthy of attention.

As the epidemic was gradually brought under control, a new autumn semester began in 2020. It was the first time that university students were welcomed back to school, but with restricted management; only those coming from regions without new positive COVID-19 patients could be approved to come back to school. School management has resulted in measures such as online and offline classes, daily reports and restriction to not to leave campus unless necessary. Previous studies have reported several cases of unbearable psychological stress among local residents living in epidemic regions and various psychological problems among university medical students (1, 3, 4). Depression symptoms, increased anxiety, and even suicidality developed among those students. It has been reported that medical staff and students were at moderate to high risk of psychological distress, among which, the detection rate of psychological distress among medical students was 30.9% (5).

COVID-19 is ongoing, and the persistent impact of the epidemic on university students, especially medical students, is not clear. Medical students are important resources for future public health. We are interested in their current mental health after they have experienced the COVID-19 outbreak and lived with COVID-19.

## 2. Background

Previous studies have shown that a professional medical career starts from the first day of entering medical school (6). Compared to students in other majors, medical students suffer more stress due to greater academic stress, more professional courses and more rigorous practical training (7). High rates of mental illness and psychological distress were found in medical students by Almojali (8) and were related to sleep disturbance. A systematic review outside North America indicated that medical students had a 7.7–65.5% prevalence of anxiety and a 6.6–66.5% prevalence of depression (9), and approximately 20.9% of Chinese medical students reported self-rated depression. The prevalence of anxiety among this group was approximately 19.6% in 2015 in Southwest China.

The mental health problems of medical school students seemed to become more common as they coped with the COVID-19 pandemic by performing social distancing (1, 10, 11). Usually, medical students need to spend a lot of time on clinical study and research in the hospital under the guidance of their mentors. Now students have to take causes online due to students learning online. They were forced to study at home and participate in distance learning. Several face-to-face tutorials, clinical clerkships and clinical exposure training had to be replaced by virtual reality tech-learning (12). These unprecedented changes and unique challenges have influenced the mental health and academic performance of medical students (13). A study found that 78.4% of medical graduate students believe that the COVID-19 pandemic has affected their studies to varying degrees (14). To some degree, medical students had to quickly adapt to new learning patterns, but some of them may not have succeeded, as everyone has their own coping style.

In the new autumn semester of 2020, most medical students returned to school and restarted their clinical clerkships. How was their mental health after experiencing quarantine at home and were now only commuting between the school and hospital? Some students may not have been able to return to school with most of their classmates, so how was their mental health?

Coping with stress is widely studied and refers to individual cognitive and behavioral strategies to master, reduce or tolerate the internal and external demands of stressful situations (4). Clinical exposure is characterized by chaos and high work demands, and future medical professionals, i.e., medical students, should be equipped with the ability to respond and react quickly and with strong resilience (15). Classen (16) reported that individuals who adopt a positive coping strategy usually have a fighting spirit and better emotional expression, which is considered to indicate better mental adjustment ability (17), which is an important competency for a health professional. Some studies have pointed out that individuals with a high degree of psychological distress will spend more time searching for information related to COVID-19, more frequently adopt a negative coping style, and report less social support (18). A number of undergraduates adopted passive strategies to deal with negative emotions, which affected their mental health (19). Compared with medical staff, the general population has a higher degree of psychological distress, and prefers to adopt negative responses to face the COVID-19 epidemic (20).

Social support, a positive factor, protects personal well-being and mental health (21). It refers to the care, love, and esteem that individuals can receive from others. Several studies have indicated that social support is a major cause of decreased negative psychological reactions such as depression and anxiety (19). It helps to decrease the harmful effects of negative events in life on physical health and emotional well-being when individuals cope with challenges (22).

The concept of stress resistance raised by Kobasa (23) showed that the influence of stressful events on human health or disease is related to individual personality traits, cognitive evaluation, coping style and social support. Research on this concept indicated that when experiencing the same stressful events, those who habitually employ a negative coping style are more likely to have psychological problems, and this concept has been convincing in nursing students in China (24). Worldwide, medical students who experienced the outbreak of COVID-19 were influenced by it.

At present, less attention has been paid to the psychological pain of medical graduate students when restricted to commuting between the campus and hospital during the COVID-19 pandemic, which might serve as the basis for the future mental health management of medical graduate students. We conducted a cross-sectional survey in Guangdong, southern China, to evaluate the incidence of anxiety and depression symptoms among postgraduate medical students; we hypothesized that there might be a mediating relationship of coping style and social support between their perceived stress and depression and anxiety symptoms. The goal is to provide targeted mental health education guidance to the graduate administration related to the hospital.

## 3. Materials and methods

### 3.1. Participants

The participants were recruited among full-time postgraduates of Southern Medical University, China. This online questionnaire survey was conducted in October 2020 and was distributed to the WeChat^®^ chat group of every class by the instructors of each grade. The inclusion criteria were as follows: (a) students over the age of 18; (b) postgraduates in any major in medicine; and (c) students with the ability to understand and complete the questionnaire. The exclusion criteria were as follows: (a) students with a history of mental illness or mental disorders; and (b) students with serious physical illness who were unable to complete the questionnaire. The respondents were informed of the purpose of the survey when they agreed to fill out the questionnaire. Only full-time medical postgraduates with the ability to understand the meaning of each question and to communicate in Chinese were included in the survey. Those who refused to participate and were unable to understand the meaning of the questions were excluded.

### 3.2. Instruments

#### 3.2.1. Demographic information questionnaire

The demographic section was designed by the research team to collect information on the general characteristics of medical postgraduates, including their gender, age, grade, place of residence, only child status, major subject, academic/professional degree and so on.

#### 3.2.2. Self-rating Depression Scale

This scale was designed in 1965 by Zung (25) and was used to measure the severity of depression. The scale includes 20 items with 10 forward and 10 reverse scoring questions. Each item is scored on a 4-point Likert scale ranging from 1 to 4 according to the frequency of symptoms in the last week. The score of each item was calculated to obtain the raw score and the standard score multiplied by 1.25, with standard scores less than 53 were viewed as indicating no depression (26), and a higher score indicated more severe depression. The Cronbach’s α coefficient was 0.89 in this study.

#### 3.2.3. Self-rating Anxiety Scale

The SAS scale was designed in 1971 by Zung (27), and it can accurately reflect the subjective feelings of patients with anxious tendencies. The scale includes 20 self-report questions with 15 forward and 5 reverse scoring questions. Each item is scored on a 4-point Likert scale ranging from 1 to 4 according to the past 7 days. The score of each item was calculated to obtain the raw score and the standard score multiplied by 1.25, with standard scores less than 50 were viewed as indicating no anxiety (26), and a higher score indicated more severe anxiety. The Cronbach’s α coefficient was 0.85 in this study.

#### 3.2.4. Questionnaire on psychological stressors of postgraduates

The scale was designed by Cheng Lina (28). The scale was compiled according to the stressors of postgraduate students with 36 items and 7 dimensions, including academic pressure, interpersonal pressure, employment pressure, family pressure, marriage pressure, economic pressure and other pressure. Using the 5-point scoring method, from the “no”, “light”, “moderate”, “heavy” and “very heavy” five aspects of the evaluation, successively recorded as 1, 2, 3, 4, 5 points. The scale has good reliability and validity and has been widely used in China (28, 29). The Cronbach’s α coefficient and split-half reliability of the scale were 0.908 and 0.86, respectively.

#### 3.2.5. Simplified coping style questionnaire

The scale was designed in 1998 by Xie Yaning (30) and contains 20 items divided into two dimensions: positive coping style (items 1–12) and negative coping style (items 13–20). The SCSQ score reflects the participant’s coping style preferences; the higher the score on the corresponding subscale is, the higher the tendency to adopt this coping style. The SCSQ scale showed good reliability and validity (31); the Cronbach’s α coefficient of the positive coping style subscale was 0.90, that of the negative coping style subscale was 0.70, and the overall Cronbach’s α coefficient of the scale was 0.85.

#### 3.2.6. Social Support Rate Scale

The scale was developed by Xiao Shuiyuan (32), a Chinese scholar at Shandong University, and was used to evaluate individuals’ social support status. It has already been widely used in different studies in Chinese communities and shown to have good validity and reliability (33, 34). It contains 10 items and is divided into 3 dimensions, including subjective support, objective support and support utilization. Subjective support reflects the individual emotional experience of being respected, supported and understood in the community. Objective support reflects objective, visible or practical support received in the past. Support utilization reflects the pattern of behavior that an individual uses when seeking social support (34). Items are scored on a 4-point Likert scale. The score was summed, generating a final score ranging from 12 to 66; the higher scores are, the stronger the social support. In this study, the total Cronbach’s α coefficient was 0.78.

### 3.3. Data analysis

Data were analyzed by SPSS 18.0 and AMOS 24.0 statistical software (IBM Inc.). Two independent sample *t*-tests were used to compare the differences in anxiety, depression, psychological stressors, social support and coping style by the binary variables, such as gender, place of residence and only child status. One-way ANOVA was used to explore the differences in anxiety, depression, psychological stressors, social support and coping style by the polytomous variables, such as age and grade. Pearson correlation was conducted to examine the relationships among depression, anxiety, psychological stressors, social support and coping style. Structural equation modeling (SEM) was conducted to assess the mediation model. The mediation effects were considered significant if the confidence intervals did not include the value of 0, and *P* < 0.05 was considered statistically significant.

## 4. Results

### 4.1. Participant characteristics

We invited 1,859 postgraduate students to fill in the questionnaire, but only 1,647 students completed all items, which also included 2 invalid questionnaires. Thus, the effective response rate is 88.5%. A total of 61.4% of the participants (*n* = 1010) were female, and approximately half of the postgraduates (51.5%) were ≤24 years old (*n* = 847). 47.3% (*n* = 778) postgraduates came from urban regions, and the rest came from rural regions. Postgraduates from the doctor grade accounted for 17.3% (*n* = 285). The first-year master’s students accounted for most of the master’s grade (*n* = 797, 48.4%).

### 4.2. Description of self-rated anxiety, self-rated depression, coping style, and social support

According to the scoring criteria, 356 (21.6%) medical postgraduates self-reported having depression symptoms, and 155 (9.4%) medical postgraduates self-reported having anxiety symptoms. The mean positive coping score was 20.32 ± 6.76, and the mean negative coping score was 7.04 ± 3.56. The total score for social support was 38.79 ± 6.75, that for subjective support was 21.76 ± 3.96, that for objective support was 8.82 ± 2.35, and that for utilization of support was 8.21 ± 2.02 (Table 1).

**TABLE 1:** Demographic characteristics and the Self-rating Depression Scale (SDS) and Self-rating Anxiety Scale (SAS) scores among medical postgraduates (mean ± SD).

Variables	*N* (%)	SDS	SAS	Positive coping	Negative coping	Social support
**Gender**						
Male	635 (38.6%)	42.49 ± 11.6*	36.63 ± 9.1	20.23 ± 7.28	6.84 ± 3.84	38.41 ± 7.3
Female	1010 (61.4%)	44.21 ± 11.09	37.2 ± 8.75	20.38 ± 6.42	7.16 ± 3.38	39.02 ± 6.37
**Age**						
≤24	847 (51.5%)	44.0 ± 10.8	37.17 ± 8.43	20.35 ± 6.56	7.15 ± 3.6	38.24 ± 6.34*
25–29	706 (42.9%)	43.27 ± 11.82	36.8 ± 9.26	20.31 ± 6.89	6.94 ± 3.5	39.2 ± 7.14
≥30	92 (5.6%)	41.52 ± 11.86	36.52 ± 10.15	20.15 ± 7.65	6.763 ± 77	40.61 ± 6.85
**Place of residence**						
Urban	778 (47.3%)	43.24 ± 11.55	36.73 ± 9.44	20.94 ± 6.98*	7.10 ± 3.64	38.63 ± 6.96
Rural	867 (52.7%)	43.83 ± 11.1	37.2 ± 8.37	19.77 ± 6.52	6.98 ± 3.5	38.92 ± 6.56
**Only child**						
Yes	488 (29.7%)	43.08 ± 11.47	36.73 ± 9.13	21.29 ± 7.04*	7.02 ± 3.4	37.9 ± 6.95*
No	1157 (70.3%)	43.75 ± 11.25	37.08 ± 8.79	19.91 ± 6.6	7.04 ± 3.63	39.16 ± 6.63
**Grade**						
Master of first grade	797 (48.4%)	42.99 ± 10.18*	36.7 ± 8.01	20.39 ± 6.11	6.93 ± 3.46	38.42 ± 6.37
Master of second grade	278 (16.9%)	45.27 ± 12.63	37.44 ± 10.03	20.34 ± 6.24	7.53 ± 3.98	38.8 ± 6.86
Master of third grade	285 (17.3%)	44.07 ± 12.53	37.13 ± 9.73	19.96 ± 7.31	6.84 ± 3.67	39.22 ± 7.28
Ph.D. student	285 (17.3%)	42.93 ± 11.56	37.17 ± 9.2	20.47 ± 7.13	7.05 ± 3.29	39.37 ± 7.09

*P < 0.05.

### 4.3. Differences in study variables among postgraduates with different characteristics

Female postgraduates had a higher SDS score than male postgraduates (44.21 ± 11.09 vs. 42.49 ± 11.6, *P* < 0.05). Postgraduates from the master of second grade had the highest SDS score (45.27 ± 12.63).

Postgraduates who lived in urban cities were more willing to positively cope with stress (*P* < 0.05). Only child postgraduates preferred a positive coping style (21.29 ± 7.04 vs. 19.91 ± 6.6, *P* < 0.05), with less social support than those who had a sister or brother (37.9 ± 6.95 vs. 39.16 ± 6.63, *P* < 0.05). Postgraduates ≤ 24 years old had the least social support (38.24 ± 6.34).

Postgraduates from different age groups experienced employment pressure, academic pressure and family pressure (*P* < 0.05). Postgraduates ≤ 24 years old had the highest employment pressure (2.39 ± 0.84), highest academic pressure (2.42 ± 0.65) and highest family pressure (1.92 ± 0.51). Postgraduates from different grades had employment pressure, academic pressure, interpersonal pressure and other pressure (*P* < 0.05). The master of third grade had the highest employment pressure (2.50 ± 0.85), and the master of second grade had the highest academic pressure (2.50 ± 0.69), highest interpersonal pressure (1.62 ± 0.69) and highest other pressure (1.79 ± 0.83) (Table 2).

**TABLE 2:** Comparison of different characteristic stressors among medical postgraduates.

Variables	*N* (%)	Employment pressure	Academic pressure	Economic pressure	Family pressure	Marriage pressure	Interpersonal pressure	Other pressure
**Gender**								
Male	635 (38.6%)	2.36 ± 0.90	2.37 ± 0.68	2.04 ± 0.63	1.9 ± 0.52	1.53 ± 0.72	1.52 ± 0.64	1.63 ± 0.74
Female	1010 (61.4%)	2.38 ± 0.83	2.43 ± 0.64	1.96 ± 0.59	1.89 ± 0.5	1.24 ± 0.49	1.48 ± 0.581	1.67 ± 0.73
**Age**								
≤24	847 (51.5%)	2.39 ± 0.84*	2.42 ± 0.65*	1.98 ± 0.6	1.92 ± 0.51*	1.34 ± 0.57	1.51 ± 0.59	1.64 ± 0.0.7
25–29	706 (42.9%)	2.39 ± 0.87	2.40 ± 0.66	2.02 ± 0.61	1.88 ± 0.5	1.36 ± 0.65	1.49 ± 0.63	1.68 ± 0.0.77
≥30	92 (5.6%)	1.98 ± 0.83	2.24 ± 0.65	1.90 ± 0.6	1.78 ± 0.56	1.35 ± 0.6	1.38 ± 0.49	1.54 ± 0.7
**Place of residence**								
Urban	778 (47.3%)	2.30 ± 0.86	2.33 ± 0.67	1.87 ± 0.59	1.83 ± 0.49	1.33 ± 0.6	1.44 ± 0.59	1.61 ± 0.73
Rural	867 (52.7%)	2.43 ± 0.85	2.47 ± 0.63	2.11 ± 0.6	1.96 ± 0.51	1.37 ± 0.61	1.55 ± 0.61	1.69 ± 0.73
**Only child**								
Yes	488 (29.7%)	2.29 ± 0.85	2.32 ± 0.66	1.88 ± 0.6	1.84 ± 0.49	1.35 ± 0.63	1.46 ± 0.61	1.63 ± 0.76
No	1157 (70.3%)	2.40 ± 0.86	2.44 ± 0.65	2.04 ± 00.6	1.92 ± 0.51	1.35 ± 0.6	1.51 ± 0.6	1.66 ± 0.72
**Grade**								
Master of first grade	797 (48.4%)	2.31 ± 0.83*	2.40 ± 0.64*	1.97 ± 0.59	1.92 ± 0.49	1.33 ± 0.58	1.45 ± 0.56*	1.57 ± 0.65*
Master of second grade	278 (16.9%)	2.47 ± 0.91	2.50 ± 0.69	2.05 ± 0.63	1.91 ± 0.55	1.44 ± 0.68	1.62 ± 0.69	1.79 ± 0.83
Master of third grade	285 (17.3%)	2.50 ± 0.85	2.41 ± 0.69	2.0 ± 0.6	1.89 ± 0.53	1.31 ± 0.54	1.55 ± 0.64	1.72 ± 0.79
Ph.D. student	285 (17.3%)	2.32 ± 0.88	2.32 ± 0.62	2.01 ± 0.62	1.83 ± 0.48	1.34 ± 0.66	1.46 ± 0.57	1.68 ± 0.76

*P < 0.05.

### 4.4. Correlation analysis between self-rated depression, self-rated anxiety, psychological stress, coping style and social support in medical postgraduates

Correlation analysis showed that self-rated depression and self-rated anxiety were positively correlated with negative coping style (*r* = 0.251, *r* = 0.275, *P* < 0.01) and psychological pressure (*r* = 0.585, *r* = 0.579, *P* < 0.01) and negatively correlated with positive coping style (*r* = −0.586, *r* = −0.467, *P* < 0.01) and social support (*r* = −0.484, *r* = −0.403, *P* < 0.01). Social support was negatively correlated with psychological pressure (*r* = −0.459, *P* < 0.01) (Table 3).

**TABLE 3:** Correlation analysis of the main indicated variables in medical postgraduates.

	Self-rated depression	Self-rated anxiety	Positive coping	Negative coping	Social support	Postgraduate pressure	Mean ± SD
Self-rated depression	1						43.41 ± 11.30
Self-rated anxiety	0.822**	1					36.86 ± 8.90
Positive coping	-0.586**	-0.467**	1				20.32 ± 6.77
Negative coping	0.251**	0.275**	0.092**	1			7.04 ± 3.57
Social support	-0.484**	-0.403**	0.464**	-0.159**	1		38.79 ± 6.75
Postgraduate pressure	0.585**	0.579**	-0.348**	0.384**	−0.459**	1	2.40 ± 0.66

**P < 0.01.

### 4.5. Mediating effects of coping style and social support on the relationships among psychological stress, self-rated depression, and self-rated anxiety

**FIGURE 1:**
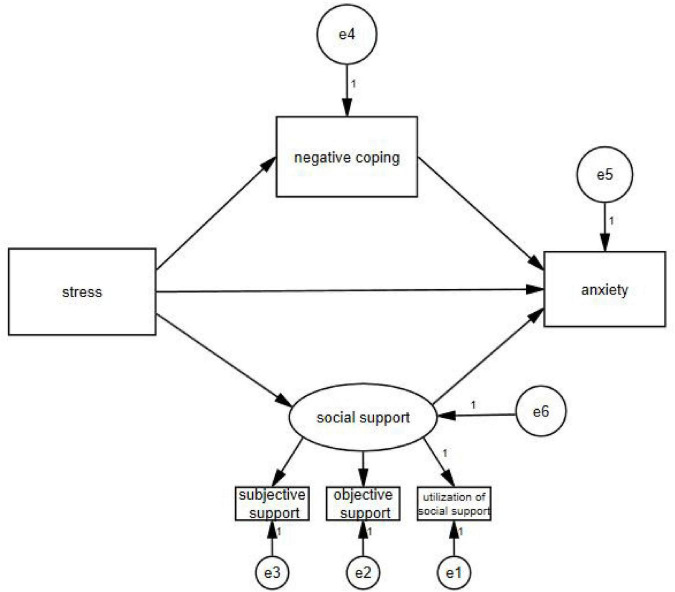
Final structural equation model on anxiety. e1–e6: the sign of the measurement error.

**FIGURE 2:**
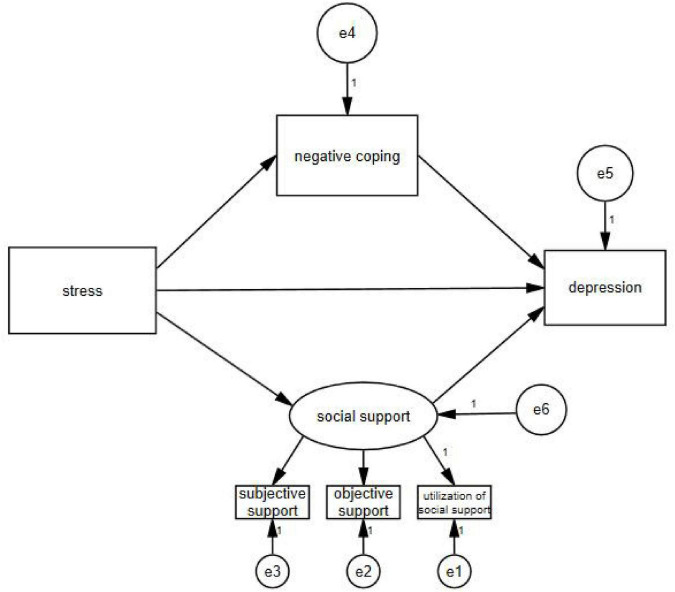
Final structural equation model on depression. e1–e6: the sign of the measurement error.

AMOS 24.0 software (IBM Inc.) was used to test and amend a hypothesized model using the maximum likelihood method of evaluating parameters. When self-rated anxiety was taken as the endogenous variable, the final model (Figure 1) had a satisfactory fit [*x^2^/df* = 3.397, goodness-of-fit index (*GFI*) = 0.995, normed fit index (*NFI*) = 0.990, incremental fit index (*IFI*) = 0.993, comparative fit index (*CFI*) = 0.993, and root mean square error of approximation (*RMSEA*) = 0.038]. When self-rated depression was taken as the endogenous variable, the final model (Figure 2) had a satisfactory fit [*x^2^/df* = 3.259, *GFI* = 0.995, *NFI* = 0.990, *IFI* = 0.993, *CFI* = 0.993, and *RMSEA* = 0.037].

Path analysis indicated that psychological stress had a direct effect on anxiety (β = 0.416, *P* < 0.01) and depression (β = 0.362, *P* < 0.01). At the same time, psychological stress, which was mediated by negative coping (β = 0.027, *P* < 0.01; β = 0.016, *P* < 0.01) and social support (β = 0.124, *P* < 0.01; β = 0.193, *P* < 0.01), also had indirect effects on self-rated anxiety and self-rated depression (Table 4).

**TABLE 4:** The total, direct and indirect effects of the exogenous variables on the endogenous variables.

Endogenous variables	Effect decomposition	Exogenous variables		
		Postgraduate pressure	Negative coping	Social support
Anxiety	Total effect	0.567	0.071	-0.236
	Direct effect	0.416	0.071	-0.236
	Indirect effect	0.151	0.000	0.000
Depression	Total effect	0.571	0.043	-0.366
	Direct effect	0.362	0.043	-0.366
	Indirect effect	0.209	0.000	0.000

Total effect = direct effect + indirect effect.

## 5. Discussion

Medical postgraduates who experienced the COVID-19 outbreak and returned to school life for the first time were in a relatively negative mood, feeling anxious and depressed. Similar to studies reporting the psychological situation of medical students worldwide (35, 36), our study also showed that self-rated anxiety and depression were high. Female students were significantly more influenced than male students, and they had lower social support. Previous findings (37) illustrated that social networks are important to personal wellbeing and mental health and that people who are socially isolated and receive less social support are more likely to develop mental health problems. After the outbreak of COVID-19, school campus lockdown and restrictions on students leaving campus have been common. Beginning a new semester after such a pandemic is a new experience, and campus management may not be tailored to every student. More friendly and attentive services and psychological education or consultation should be arranged in universities that are locked down, especially for those who are isolated, who may feel more burnout and psychological distress.

Perceived stress, such as employment pressure, interpersonal pressure and academic pressure, was the main stressors among the medical postgraduates. Master’s students who were looking for jobs had the most difficult time since several job fairs were delayed or even canceled, as companies were also influenced by the lockdown and social isolation. Online interviews (38) were reported to be strict, and interviewees were more nervous than for offline interviews and less satisfied with the virtual activity. Due to the employment pressure among students in graduating grades, emphasis should be placed on the important tasks of university management, vocational counseling, and interview training, and job-seeking subsidies should be provided.

The 2nd-year postgraduates faced interpersonal pressure. Online classes were the only way that medical postgraduates took classes. Students spent little time on activities with their other classmates aside from their roommates. Presentations and group seminars were held through a computer screen, as a kind of telecommunication. This was reported to be burdensome, leading to exhaustion and anxiety (39). Some surveys reported that medical students were not satisfied with online learning, while others reported that students had more control over educational content (40). Some students were working in the hospital, wearing masks in the ward, inpatients and outpatient clinics. A survey reported that people had difficulty communicating with masks (41). Their voice had to be raised, and the mask could cause sweating. The presence of only eye and vocal contact may not be persuasive when a trainee doctor communicates with his or her patients.

The master of second grade experienced high academic pressure. Medical postgraduates usually start their thesis proposal in the 2nd year, and several projects should be completed in the hospital. Although these students were not locked down on the university campus, their decreased interchange with patients, disruptions to medical care and stricter ward management made the thesis study process slow. Moderate-to-high academic stress (42) was reported to be quite common among college students during the COVID-19 pandemic, affecting their study attitudes.

Studying medicine has highly professional demands and academic requirements (43). Appropriate coping and support from family, classmates and universities could mediate personal perceived stress. In our study, coping style and social support played a mediating role between perceived stress and anxiety/depression. Positive coping style and social support reduced the impact of perceived stress on depression and anxiety, while negative coping strengthened the impact of perceived stress on depression and anxiety. Receiving sufficient social support or engaging in positive coping could help individuals manage depression and anxiety.

The unpredictable nature of the COVID-19 epidemic reshaped the social order and daily routines. The ability to cope with uncertainty and engage in positive coping were basic abilities that doctors and citizens living with the COVID-19 pandemic should have (15, 44). Medical postgraduates face more pressure than undergraduates, and in the literature on medical students, self-care ability, self-efficacy and optimism have been shown to be important to their resilience. Many of these postgraduates will become part of the medical health workforce, and we suggest that more theory-based interventions and resilience strengthening be performed during the epidemic. Perhaps we can carry out psychological interventions, such as cognitive behavioral therapy, based on the characteristics of medical graduate students. During the COVID-19 pandemic, online psychosocial interventions can effectively improve individual symptoms of anxiety, depression, and stress (45).

## 6. Conclusion

This study successfully explored the mental health impact of the COVID-19 pandemic on current medical postgraduates restricted to campus hospital studies. The results showed that postgraduates troubled by employment, academic and interpersonal stress were more likely to have anxiety and depression, and may need further mental health support and learning guidance. As COVID-19 continues, the social support and guiding the positive coping of the medical graduate students will need to be strengthened in the future to alleviate their perceived stress and negative emotions.

## 7. Limitations

Our study focused on the metal health of medical postgraduates and we found that pressure and negative coping style were risk factors to depression and anxiety. However, this study is a cross-sectional study, which cannot reveal the causal direction of the relationship between anxiety, depression, social support, and coping styles. Future prospective cohort studies are needed to explore the long-term psychological impact of COVID-19 on medical postgraduates. In addition, relevant factors that might influence the findings of the study, such as student’s field of study, family/social support were not investigated in the current study. Future studies are needed to determine the effect of these confounders on medical student’s psychological distress.

## Data availability statement

The original contributions presented in this study are included in this article/supplementary material, further inquiries can be directed to the corresponding author/s.

## Ethics statement

The studies involving human participants were reviewed and approved by the Biomedical Ethics Committee of Southern Medical University. Written informed consent for participation was not required for this study in accordance with the national legislation and the institutional requirements.

## Author contributions

GH and YC: study design. TT, QZ, and YW: data collection, analysis, and interpretation. TT, WL, QZ, and YH: drafting of the manuscript. YY and XW: critical revision of the manuscript. All authors contributed to the article and approved the submitted version.
